# Supplementary material for: De novo DNA methyltransferase activity in colorectal cancer is directed towards H3K36me3 marked CpG islands
Source: Nat Commun. 2021 Jan 29;12:694. doi: 10.1038/s41467-020-20716-w (PMC7846778; doi:10.1038/s41467-020-20716-w)
Supplement: Supplementary file 1 — Supplementary Information [file 41467_2020_20716_MOESM1_ESM.pdf]

Supplementary Tables

Supplementary Table 1

Clone statistics for bisulphite PCR analysis of CGIs at native and ectopic loci in HCT116 cells.

CGI	Total number of clones	Number of clones by PCR amplicon
Native locations		
BUB1	7	L: 3, R: 4
MLH1	7	L: 7
CDKN2A	23	L: 21, R:2
SFRP1	18	L: 10, R: 8
ZFP42	16	L: 10, R: 6
GATA4	25	L: 16, R: 9
CDH7	41	L: 19, R1: 17, R2: 5
CDH13	30	L: 11, R1: 3, R2: 16
EPHB1	20	R: 20
DAZL	25	L: 9, R: 16
Ectopic locations		
BUB1	10	L1: 10
MLH1	15	L: 6, R: 9
CDKN2A	13	L: 6, R: 7
SFRP1	12	R1: 6, R2: 6
ZFP42	7	L: 2, R:5
GATA4	8	L1: 3, L2: 3, R: 2
CDH7	34	L: 4, R1: 14, R2: 16
CDH13	24	L: 7, R1: 10, R2: 7
EPHB1	11	L: 1, R1: 4, R2: 6
DAZL	22	L: 8, R: 14

Supplementary Table 2

Clone statistics for bisulphite PCR analysis of CGIs at native and ectopic loci in RKO cells.

CGI	Total number of clones	Number of clones by PCR amplicon
Native locations		
MLH1	17	L: 17
CDKN2A	39	L: 27, R: 12
SFRP1	24	L: 13, R: 11
CDH7	30	L: 16, R2: 14
Ectopic locations		
MLH1	28	L: 22, R: 6
CDKN2A	21	L: 21
SFRP1	24	R1: 3, R2: 6
CDH7	29	L: 20, R2: 9

Supplementary Table 3
GO-terms enriched in genes associated with DNMT3B targets in DKO cells.

P-values are from Benjamini-Hochberg corrected two-sided Fisher's exact tests.
Fold change was calculated as the % of DNMT3B target CGIs associated with a gene annotated with a specific term over the % control CGIs associated with a gene annotated with that term.

Term ID	Adjusted p-value	Fold change	Term description
GO:0032502	0.001	1.127	developmental process
GO:0044767	0.001	1.130	single-organism developmental process
GO:0004672	0.004	1.544	protein kinase activity
GO:0016301	0.007	1.429	kinase activity
GO:0016773	0.007	1.436	phosphotransferase activity, alcohol group as acceptor
GO:0007389	0.007	1.445	pattern specification process
GO:0009653	0.007	1.189	anatomical structure morphogenesis
GO:0009792	0.007	1.408	embryo development ending in birth or egg hatching
GO:0043009	0.007	1.406	chordate embryonic development
GO:0044707	0.007	1.109	single-multicellular organism process
GO:0048856	0.007	1.117	anatomical structure development
GO:0071560	0.007	1.885	cellular response to transforming growth factor beta stimulus
GO:0001501	0.009	1.477	skeletal system development
GO:0003002	0.010	1.453	regionalization
GO:0007155	0.010	1.327	cell adhesion
GO:0048731	0.010	1.128	system development
GO:0048732	0.010	1.469	gland development
GO:0072364	0.010	5.681	regulation of cellular ketone metabolic process by regulation of transcription from RNA polymerase II promoter
GO:0009790	0.011	1.291	embryo development
GO:0032501	0.011	1.095	multicellular organismal process
GO:0048513	0.011	1.155	animal organ development
GO:0014902	0.014	2.209	myotube differentiation
GO:0007275	0.015	1.117	multicellular organism development
GO:0022610	0.023	1.280	biological adhesion
GO:0048568	0.023	1.394	embryonic organ development
GO:0060541	0.023	1.639	respiratory system development
GO:0060765	0.023	3.458	regulation of androgen receptor signaling pathway
GO:1903798	0.023	3.458	regulation of production of miRNAs involved in gene silencing by miRNA
GO:0071559	0.025	1.661	response to transforming growth factor beta
GO:0009798	0.025	1.961	axis specification
GO:0050704	0.025	3.550	regulation of interleukin-1 secretion
GO:0060965	0.026	4.754	negative regulation of gene silencing by miRNA
GO:0016310	0.026	1.212	phosphorylation
GO:0060323	0.026	2.768	head morphogenesis
GO:0060324	0.026	2.768	face development
GO:0070920	0.026	3.314	regulation of production of small RNA involved in gene silencing by RNA
GO:0048705	0.026	1.551	skeletal system morphogenesis
GO:0032870	0.029	1.410	cellular response to hormone stimulus
GO:0035033	0.031	7.154	histone deacetylase regulator activity
GO:0030521	0.032	2.933	androgen receptor signaling pathway
GO:0016772	0.034	1.328	transferase activity, transferring phosphorus-containing groups
GO:0060589	0.034	1.580	nucleoside-triphosphatase regulator activity
GO:0000975	0.034	1.229	regulatory region DNA binding
GO:0001067	0.034	1.228	regulatory region nucleic acid binding
GO:0008047	0.034	1.490	enzyme activator activity
GO:0030695	0.034	1.601	GTPase regulator activity
GO:0044212	0.034	1.234	transcription regulatory region DNA binding
GO:0010646	0.034	1.150	regulation of cell communication
GO:0023051	0.035	1.148	regulation of signaling
GO:0045624	0.035	3.976	positive regulation of T-helper cell differentiation
GO:0060149	0.035	3.976	negative regulation of posttranscriptional gene silencing
GO:0060967	0.035	3.976	negative regulation of gene silencing by RNA
GO:0010171	0.036	2.329	body morphogenesis
GO:0050793	0.036	1.166	regulation of developmental process
GO:1903844	0.041	1.861	regulation of cellular response to transforming growth factor beta stimulus
GO:0007179	0.042	1.853	transforming growth factor beta receptor signaling pathway
GO:0051148	0.045	2.121	negative regulation of muscle cell differentiation
GO:0065005	0.045	3.001	protein-lipid complex assembly
GO:0034399	0.045	1.949	nuclear periphery
GO:0031063	0.046	2.864	regulation of histone deacetylation
GO:0051154	0.046	2.864	negative regulation of striated muscle cell differentiation
GO:0060972	0.046	3.493	left/right pattern formation
GO:0043372	0.046	3.728	positive regulation of CD4-positive, alpha-beta T cell differentiation
GO:0072608	0.049	5.681	interleukin-10 secretion
GO:2001179	0.049	5.681	regulation of interleukin-10 secretion

Supplementary Table 4
Oligonucleotides used in this study.

Primers for cloning CGIs - includes 15bp homology for HD cloning in pEGFP-N2-pB-min vector

Target	Primer sequences (F/R)
VWA1	TTGACGCATGGCTAGGCTACCACGTGCAGTTCG / AATTGACGCATGTTAAATTATCACCCACGGACCC
TNFRSF1A	TTGACGCATGGCTAGCCGCACGAATTCCTTCCAGC / AATTGACGCATGTTACGCCACCTTCTCTCTTTCAGGG
CDH7	TTGACGCATGGCTAGTTCGCGTTCTTTTCCAGTAGCCC / AATTGACGCATGTTACGTGTGCATCCGAAAGAACGTG
CDH13	TTGACGCATGGCTAGGCCTCTACCCAATGCTTTCGTGA / AATTGACGCATGTTAAGCTCTCTCCTCCTCCCGTTAAC
CDKN2A	TTGACGCATGGCTAGGCCTCCGACCGTAACTATTCGG / AATTGACGCATGTTAGAGCCCAGTCCTCCTTCCTTG
BUB1	TTGACGCATGGCTAGCTAACGAATTATCCAGATTGCTCCA / AATTGACGCATGTTAGGCCAGGTTTCGGTTCAAC
ZFP42	TTGACGCATGGCTAGTGATTACACCCACGCGTATTTGT / AATTGACGCATGTTACATGAAACAAGACTCACCCCTGTT
EPHB1	TTGACGCATGGCTAGTAGCTAGCAATGTGACACCAGGA / AATTGACGCATGTTAGCAAAGCAGCCAGAGGAACC
DAZL	TTGACGCATGGCTAGGCCGAGTTTCACCCACGAGTGAA / AATTGACGCATGTTAGACTGAGGCACCGGACCTGC
SFRP1	TTGACGCATGGCTAGCGTCCTGCCGCAAACTTCCAG / AATTGACGCATGTTATCTCCCCTTGTCTCTTTCCTCCT
MLH1	TTGACGCATGGCTAGGACGAAGAGACCCAGCAACCCAC / AATTGACGCATGTTAAGAAACACACGGTCTGCGGAAAA
GATA4	TTGACGCATGGCTAGCGGGGAGGGAGAAAGGGAAC / AATTGACGCATGTTAGTTGGGCAATTTCGGTGAAGTGA

bsPCR primers for native CGI loci

Target	Primer sequences (F/R)
CDH7 L	AAGGAATTTATTTGTATAGATTYGTTAGG / AACTCCTCCTCCAATCACAAC
CDH7 R1	AGTGGGTTGTGATTGGAGG / CACTCACCTAAAACCTCRCTA
CDH7 R2	GTTGTGATTGGAGGAGGAGTT / CAACAAAAACRCACATCTACC
CDH13 L	TTTTGTAAAGTTATTGGTTTTGGTTATTA / CAACCCCTCTTCCCTACCT
CDH13 R1	AGGTAGGGAAGAGGGGTTG / CCTCTAACTAAATCTTTCTCCAATACA
CDH13 R2	TTTTTTGTTTTAGGTAGGGAAGAGG / AAAAACCAAAATTACCCCACTTAATATAAA
BUB1 L	ATATAAGGTTTAGGATTTTTGTTAGGTTG / ACCTACCTTCTTTTACCCCCT
BUB1 R	AGGGGGTAAAAGAAGGTAGGT / CCTAAATAACTAAAACAAAAAAACCAATTC
GATA4 L	GGATGAGGATTATAGGAAGGGG / AACCCTACCTACTAAACCTAAAAATTC
GATA4 R	GAATTTTTAGGTTTAGTAGGTAGGGTT / AAAAACAAAACAAAAATTAAACAATTTCR
ZFP42 L	GGTTTAAAAGGGTAAATGTGATTATATTTA / ATCTAATCAAACTACAACCACCCA
ZFP42 R	AGTTGATGGGTGGTTGTAGTT /ACACATTCCAAATAATAAACAACACAA
DAZL L	TGTGGGTTATGGTTGTGGTG / CTTCATCTTTAACTCCTTTAACCACT
DAZL R	AGTGGTTAAAGGAGTTAAAGATGAAG / RCCTTCCTAAAACTAAAACACC
SFRP1 L	TTTTGTYGTAAATTTTTAGGGATTTT / CACTCCAACCCTACAACCTC
SFRP1 R	GAGGTTGTAGGGTTGGAGTG / TCTAATTCTAATAAACCRAACC
EPHB1 R	GTTGTTGTTTYGGTTTGGTTT / ACTCCCAAACCTCCTTACCC
MLH1 L	GCCTCGTCGACTTCCATCTTGCT / CGAATAACCCCTGCCACGAACGA
CDKN2A L	GTTTTYGTTGTAGATTTTTTATTTATTTGGAT / ATCCCTCCAAAAAATTTAAAAAACAAAAT
CDKN2A R	ATTTTGTTTTTTAAATTTTTTGGAGGGAT / CCAATCCTCCTTCCTTACCAA
VWA1	TGTTTTTTGATTTTTTGTATTATTTATAGA / CACCAACTCCAACACATAATAACC
TNFRSF1A	GTTTTGTTGGTGAGGATATTTAAAA / CAAAAACAAACAAAAACTAACAACC
BRCA2	GGGGAATAGGTTTTGAGAGAATATT / CCAAACCACCCTACTTAAAAAAAC
MIDN1 1	ATTTGGTTTTTAGAATTATTTTTTG / AACCCCTAAACAAATCAACTACTC
MIDN1 2	GGTAYGTTATATTTTAATTGTTAAGATAGTAG / CTAACTCTAACCCTACAACAAAAAAAC

bsPCR primers for integrated CGI loci

Target	Forward primer sequence
CDH7 L	GATAGTTTGCGTAAAATTGACGTATG / TCCTCCAATCACAACCCACT
CDH7 R1	GTTGTGATTGGAGGAGGAGTT / TAAAAATAATCATACGTAAAATTAACGCATA
CDH7 R2	GTTGTGATTGGAGGAGGAGTT / TTAACCCTAAAAAAATAATCATATTATAACRTA
CDH13 L	TTAATTTTAGAAAGATAGTTTGYGTAAAATTG / CAACCCCTCTTCCCTACCT
CDH13 R1	AGGTAGGGAAGAGGGGTTG / TAAAAATAATCATACGTAAAATTAACGCATA
CDH13 R2	AGGTAGGGAAGAGGGGTTG / TTAACCCTAAAAAAATAATCATATTATAACRTA
ZFP1 L	GATAGTTTGCGTAAAATTGACGTATG / ATCTAATCAAACTACAACCACCCA
ZFP1 R	TGGGTGGTTGTAGTTTGATTAGAT / TAAAAATAATCATACGTAAAATTAACGCATA
GATA4 L1	GATAGTTTGCGTAAAATTGACGTATG / AACCCTACCTACTAAACCTAAAAATTC
GATA4 L2	TTAATTTTAGAAAGATAGTTTGYGTAAAATTG / CTCCCCCAACAAACAAAATCC
GATA4 R	GAATTTTTAGGTTTAGTAGGTAGGGTT / TAAAAATAATCATACGTAAAATTAACGCATA
EPHB1 L	GATAGTTTGCGTAAAATTGACGTATG / ACACCAACAACCAACTTACTC
EPHB1 R1	GGTTTTGGATTATTTATTATTGTTTTTTTTGG / GATAGTTTGCGTAAAATTGACGTATG
EPHB1 R2	GTTGTTGTTTYGGTTTGGTTT / TAAAAATAATCATACGTAAAATTAACGCATA
SFRP1 R1	GAGGTTGTAGGGTTGGAGTG / TAAAAATAATCATACGTAAAATTAACGCATA
SFRP1 R2	GTATTGATTTYGGAGGTTGTAGG / TAAAAATAATCATACGTAAAATTAACGCATA
DAZL L	GATAGTTTGCGTAAAATTGACGTATG / CCTCACAACAACCCCAAAAATAA
DAZL R	TTATTTTTGGGGTTGTTGTGAGG / TAAAAATAATCATACGTAAAATTAACGCATA
MLH1 L	GATAGTTTGCGTAAAATTGACGTATG / ATCATCTCTTTAATAACATTAACTAACC
MLH1 R	GGTTAGTTAATGTTATTAAAGAGATGA / TAAAAATAATCATACGTAAAATTAACGCATA
CDKN2A L	GATAGTTTGCGTAAAATTGACGTATG / ATCCCTCCAAAAAATTTAAAAAACAAAAT
CDKN2A R	TAGTTAGTYGAAGGTTTTATGTTGTTT / TAAAAATAATCATACGTAAAATTAACGCATA
BUB1 L	GATAGTTTGCGTAAAATTGACGTATG / CCCCAAACCTACCTTCTTTTAC

Primers for Sanger sequencing of pGEM T-Easy bisulfite clones

Target	Primer sequence
T7	TAATACGACTCACTATAGGG
SP6	TATTTAGGTGACACTATAG

ChIP primers for CGI loci

Target	Forward primer sequence
VWA1	AACTGACCGCTGTTCCCTGA / GCACCAGCTCCAGCACATAG
TNFRSF1A	TGAAAAAGGCTCAGGGACGA / CGAGGATGAGGGACGCTATG
BRCA2	AGTGGTGGTGGTAGTGGGTTGG / TTGGCAGAGACAAAAGGGCAAG
MIDN1	GCCCACATGTCCACGCCATTGC / GTTGGGGTGTAGCGTGCCTGC
MAP1S	TGGTGTCACCCTGTGAATTTGAGC / CACGGGATCCGAGTCAGACAGG

Oligonucleotides for plasmid construction

Oligo name	Sequence
Min-req-T oligo	ATGCATTAGTTATTATTAACCCTAGAAAGATAGTCTGCGTAAAATTGACGCATGGCTAGCACCGGTACTAGTTTAATTAACATGCGTCAATTTTACGCATGATTATCTTTAACGTACGTCACAATATGATTATCTTTCTAGGGTTAATAATAGTAATCAATT
Min-req-B oligo	AATTGATTACTATTATTAACCCTAGAAAGATAATCATATTGTGACGTACGTTAAAGATAATCATGCGTAAAATTGACGCATGTTAATTAAACTAGTACCGGTGCTAGCCATGCGTCAATTTTACGCAGACTATCTTTCTAGGGTTAATAATAACTAATGCAT
DNMT3B-KI-gRNA24_T	CACCGAAAGCATGAAGGGAGACACC
DNMT3B-KI-gRNA24_B	AAACGGTGTCTCCCTTCATGCTTTC
U-3B-3XT7-5'	TCAGAATTCCACCCTAAGAATGCATCCTGGGGCCTTGGCCTGAGAACACAGAGCCCATGGCGGGGGAACACTGTCCCTCTCATGTCCCTGCTTCCCTTTCACCCACCCATTCTGGCTTCTCCCACAGGAAAGCATGGCTTCCATGACCGGCGGGCAGC
U-3XT7	ATGGCTTCCATGACCGGCGGGCAGCAAATGGGCGGGTCCAGCGCCAGCATGGCCTCTATGACAGGGGGACAACAGATGGGGGGAAGCTCTGCTAGAATGGCTAGCATGACAGGAGGCCAGCAAATGGGATCTAGA
U-3B-3XT7-3'	ATCGAATTCCGGATAGCCTCCAGGATTGGGGGCGAGTCGGAGGACTGGTCGCTGCAGGCCCCGTTGACGAGGATCGAGTCTTCCCTCCCGCCGGCGTCCTCTTCTCCATTGAGATGTCTCGTGTCTCCCTTCATTCTAGATCCCATTTGCTGGCCTCCT
gDNA-3XT7-3B_T	CCTGTCCACATGGAACCAAGTCC
gDNA-3XT7-3B_B	CTGTTGTCCCCCTGTCATAGAGG

Supplementary Table 5

Summary of sequencing statistics for RRBS. Aligned reads counts are following PCR duplicate removal.

Sample	Total reads ($x10^6$)	Aligned reads ($x10^6$)	Mean CG coverage	Bisulfite conversion rate
DNMT3B expression in DKO cells experiment 1				
HCT116	57.98	31.22	26.83	99.67%
DKO	56.16	30.61	26.53	99.69%
DKO + DNMT3B	56.12	32.21	29.10	99.66%
DKO + DNMT3Bcd	52.63	28.31	25.19	99.62%
DNMT3B expression in DKO cells experiment 2				
HCT116	38.46	22.68	20.30	99.67%
DKO	42.46	25.46	23.03	99.68%
DKO + DNMT3B	46.99	28.40	25.83	99.68%
DKO + DNMT3Bcd	46.42	27.87	25.42	99.68%
DKO + GFP	80.56	48.64	42.78	99.68%
5-aza-dC treatment of HCT116				
HCT116 day 0	As HCT116 in DKO experiment 1			
HCT116 day 3	60.98	30.80	25.59	99.65%
HCT116 day 6	50.83	26.75	23.17	99.68%
HCT116 day 13	49.68	28.41	25.50	99.65%
HCT116 day 16	61.50	32.86	27.84	99.66%
HCT116 day 22	52.88	28.29	24.21	99.67%
HCT116 day 28	60.14	33.91	30.37	99.67%
HCT116 day 40	53.64	28.12	23.94	99.67%
HCT116 day 40 control	47.91	25.66	21.76	99.67%
5-aza-dC treatment of DNMT3B KO				
DNMT3B KO day 0	48.41	27.88	25.04	99.66%
DNMT3B KO day 3	47.92	26.99	23.64	99.67%
DNMT3B KO day 6	45.87	23.22	20.43	99.68%
DNMT3B KO day 13	59.87	31.94	26.78	99.67%
DNMT3B KO day 16	49.27	27.42	23.96	99.69%
DNMT3B KO day 22	53.32	29.40	25.45	99.69%
DNMT3B KO day 28	51.53	28.62	24.49	99.67%
DNMT3B KO day 40	47.75	26.36	23.25	99.70%
DNMT3B KO day 40 control	54.05	30.80	27.42	99.69%
5-aza-dC treatment of DNMT1 KO				
DNMT1 KO day 0	61.57	33.50	28.23	99.66%
DNMT1 KO day 3	51.79	26.85	23.45	99.68%
DNMT1 KO day 6	48.56	25.17	22.35	99.67%
DNMT1 KO day 13	56.15	29.72	25.92	99.68%

DNMT1 KO day 16	49.88	27.04	23.28	99.66%
DNMT1 KO day 22	59.70	32.34	27.56	99.67%
DNMT1 KO day 40	52.22	27.10	23.67	99.68%
DNMT1 KO day 40 control	55.88	31.63	28.30	99.67%

Supplementary Table 6

Summary of sequencing statistics for ChIP-Rx-seq. Aligned reads are after duplicate and multi- mapper removal and reported for both genomes (hg38/dm6). For T7-DNMT3B ChIP-Rx-seq, which was paired end sequencing, fragments are quoted.

Sample	total reads or read pairs (x10^6)	Aligned reads or fragments hg38/dm6 (x10^6)
	H3K36me3	
HCT116 Input rep. 1	70.54	59.19/0.11
HCT116 H3K36me3 rep. 1	70.07	58.09/0.29
DKO Input rep.1	72.20	60.76/0.12
DKO H3K36me3 rep. 1	62.24	55.08/0.22
HCT116 Input rep. 2	72.15	60.51/0.13
HCT116 H3K36me3 rep. 2	70.96	58.78/0.28
DKO Input rep.2	74.59	62.69/0.14
DKO H3K36me3 rep. 2	72.79	60.51/0.24
	T7-DNMT3B	
HCT116 Input rep. 1	74.80	63.42/0.005
HCT116 T7-IP rep. 1	52.35	39.36/1.902
T7-DNMT3B Input rep. 1	78.31	66.45/0.005
T7-DNMT3B T7-IP rep. 1	73.32	62.90/0.332
HCT116 Input rep. 2	74.94	63.58/0.006
HCT116 T7-IP rep. 2	66.10	56.25/0.085
T7-DNMT3B Input rep. 2	68.89	57.81/0.006
T7-DNMT3B T7-IP rep. 2	83.90	70.50/0.179

Supplementary Figure 1

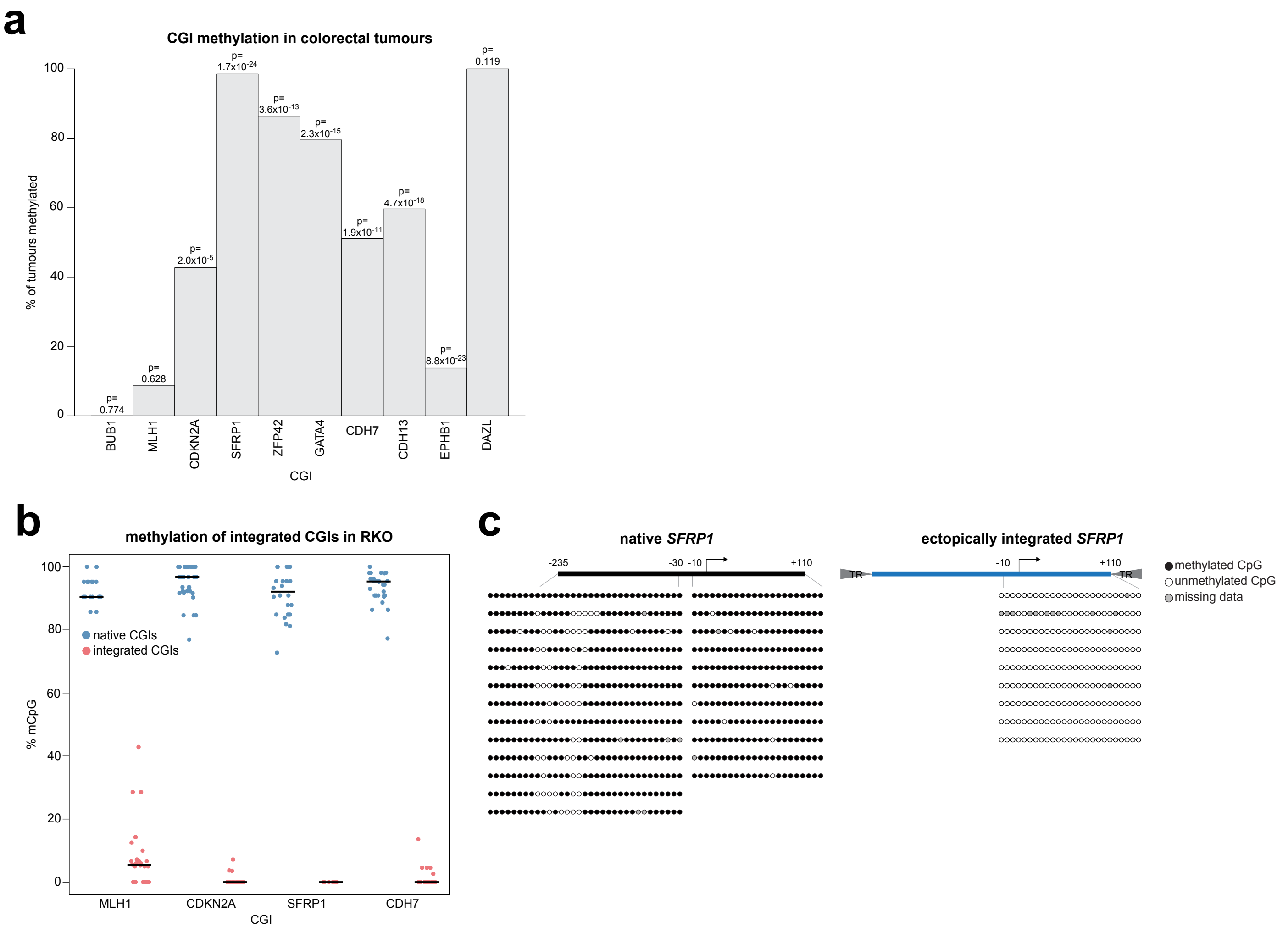

Supplementary Figure 1

CGIs are not de novo methylated at ectopic locations in colorectal cancer

a) Frequency of methylation at CGIs integrated using piggyBac in 342 colorectal tumours. P-values were derived from comparing mean methylation at these CGIs in the colorectal tumours to that observed in 42 normal samples (two-sided Wilcoxon rank sum tests). b) Ectopically integrated aberrantly methylated CGIs do not become de novo methylated in RKO cells. Plot showing the mean methylation per clone as assayed by bsPCR for 4 CGIs in their native location and when ectopically integrated in a piggyBac transposon. Each point represents the mean methylation level for a single bsPCR clone. The thick line indicates the median for each CGI. The number of clones analysed per CGI is indicated in Supplementary Table 2. c) *SFRP1* is not hypermethylated when integrated into ectopic locations in RKO cells. Illustrative example bsPCR data from panel *b* showing the *SFRP1* promoter CGI in its native and integrated state. Circles are CpGs with different clones arranged vertically. Each integrated clone derives from a separate genomic integration. Black circles are methylated CpGs and white circles are unmethylated CpGs. Grey circles represent missing data due to sequencing errors. Source data are provided as a Source Data file.

Supplementary Figure 2

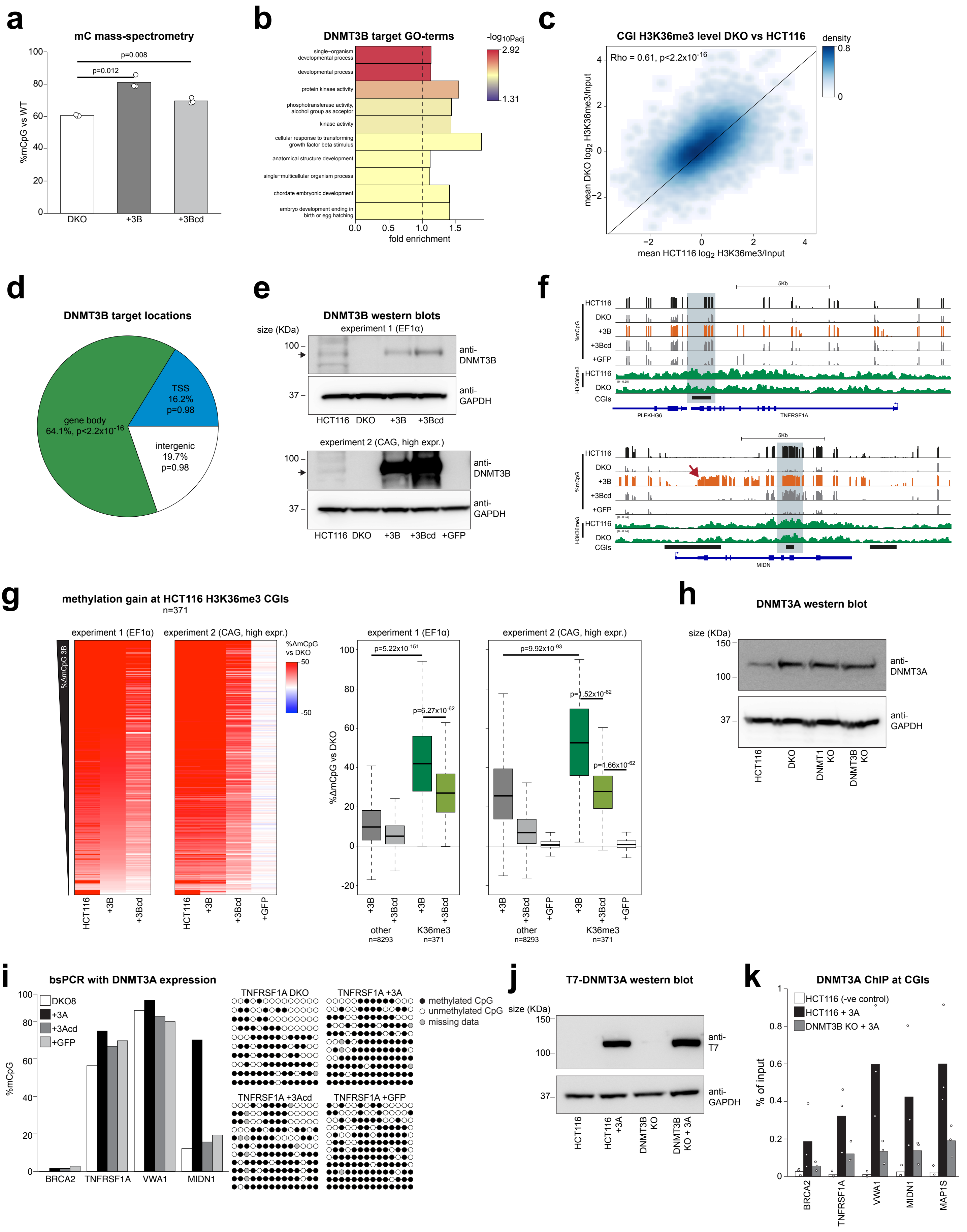

Supplementary Figure 2
DNMT3B methylates H3K36me3 marked CGIs in colorectal cancer cells
a) Expression of DNMT3B in DKO cells results in a global gain of DNA methylation. Barplot of total methylated cytosine levels estimated by mass-spectrometry. Shown are mean methylation levels relative to HCT116 cells from 3 technical replicates. P-values are from two-sided t-tests. +3B = DKO + DNMT3B; +3Bcd = DKO + catalytically dead DNMT3B. b) Gene ontology analysis of genes associated with DNMT3B target CGIs. Top ten significant hits shown. Colour indicates Benjamini-Hochberg adjusted FDR significance level, DNMT3B target CGIs (n = 2,238) versus all CGIs (n = 22,179), two-sided Fisher's exact tests. Full list of significant GO-terms is included in Supplementary table 3. c) H3K36me3 levels at CGIs in DKO cells are correlated to those in HCT116 cells. Density scatter plot of mean normalised H3K36me3 levels at CGIs in DKO cells versus those in HCT116 cells (2 biological replicates each, n= 23,715 CGIs). Spearman's Rho correlation is indicated. P-value from Spearman's correlation test. d) DNMT3B target CGI are enriched in gene bodies. Pie chart showing the percentage of DNMT3B target CGIs in each category. P-values from one sided Fisher's exact tests for enrichment, DNMT target CGIs (n = 2,238) versus all CGIs (n = 22,179). e) Western blots comparing DNMT3B expression levels in two experiments. The upper panels show data from the first experiment (data in Figure 2) where the EF-1α promoter drives DNMT3B expression. The lower panels show data from the second experiment where the CAG promoter drives higher levels of DNMT3B. Arrows indicate DNMT3B. +3B = DKO + DNMT3B; +3Bcd = DKO + catalytically dead DNMT3B; +GFP = DKO + GFP. f) Examples of DNMT3B target CGIs from the second experiment. Genome browser plots showing DNA methylation levels with HCT116 and DKO H3K36me3 ChIP signal. CGIs and genes are shown below the plots. CGIs gaining methylation when DNMT3B is expressed in DKO cells are indicated in light blue. +3B = DKO + DNMT3B; +3Bcd = DKO + catalytically dead DNMT3B; +GFP = DKO + GFP. Scale for methylation data is 0 to 100%. For H3K36me3 it is 0 to 0.24 normalised reads per 10^6. g) H3K36me3 marked CGIs gain methylation when DNMT3B is expressed in DKO cells. Left, heatmaps of relative methylation levels at methylated HCT116 H3K36me3 marked CGIs. Values denote the change in methylation relative to DKO cells. CGIs are ranked by their mean gain of methylation in experiment 1. Left, boxplots of relative methylation at H3K36me3 marked CGIs and all other CGIs methylated in HCT116 cells. P-values are from two-sided Wilcoxon rank sum tests. Lines=median; box=25th–75th percentile; whiskers=1.5× interquartile range from box. h) DNMT3A is upregulated in DNMT knockout cells. Western blot for DNMT3A in HCT116 cells, DKO cells and HCT116 cells lacking either DNMT1 (DNMT1 KO) or DNMT3B (DNMT3B KO). This experiment was repeated twice with similar results. i) Overexpression of DNMT3A in DKO cells results in gains of DNA methylation at DNMT3B targets. Left, barplot showing mean methylation levels estimated by bisulfite PCR at CGIs in DKO8 and transfected DKO cells. Right, representative example bsPCR data from the TNFRSF1A gene CGI. +3A = DKO + DNMT3A; +3Acd = DKO + catalytically dead DNMT3A; +GFP = DKO + GFP. Circles are CpGs with different clones arranged vertically. Black circles are methylated CpGs and white circles are unmethylated CpGs. Grey circles represent missing data due to sequencing errors. j) Ectopic T7-DNMT3A is expressed to a similar level in HCT116 and DNMT3B KO cells. Western blot for T7-tagged DNMT3A in HCT116 cells and

HCT116 cells lacking DNMT3B (DNMT3B KO). This experiment was conducted once. k) DNMT3A is recruited to the genome more efficiently in the presence of DNMT3B. qPCR analysis of CGIs following ChIP for T7-tagged DNMT3A in HCT116 and DNMT3B KO cells expressing T7-DNMT3A. The bars indicate the mean of 3 biological replicates with the individual replicates shown as points. Source data are provided as a Source Data File. Uncropped western blots for panels e, h and j are supplied in Supplementary Figure 7.

Supplementary Figure 3

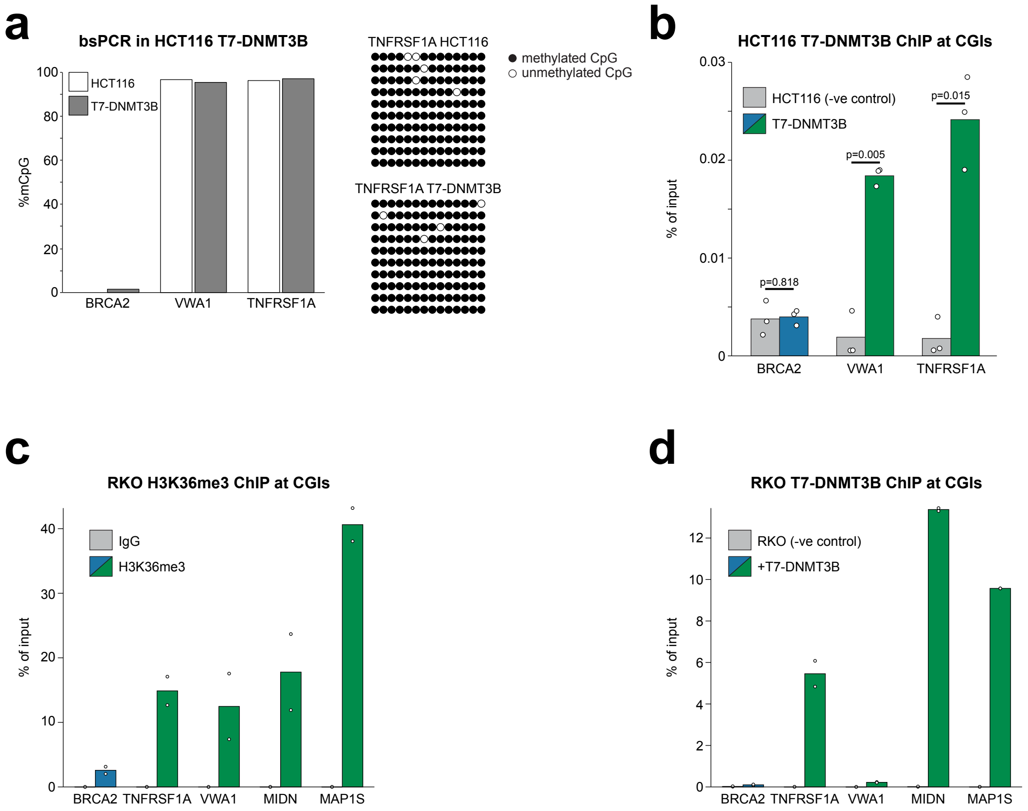

Supplementary Figure 3

DNMT3B is recruited to H3K36me3 marked CGIs

a) T7 tagging of endogenous DNMT3B does not result in losses of DNA methylation at CGIs. Left, barplot showing mean methylation levels estimated by bisulfite PCR at CGIs in HCT116 and T7-DNMT3B cells. Right, representative example bsPCR data from the *TNFRSF1A* gene CGI. Circles are CpGs with different clones arranged vertically. Black circles are methylated CpGs and white circles are unmethylated CpGs. b) DNMT3B localises to CGIs that gain methylation upon DNMT3B expression in DKO cells. qPCR analysis of CGIs following anti-T7 ChIP from T7-DNMT3B cells and HCT116 cells lacking the tag (-ve control). The bars indicate the mean of 3 biological replicates with the individual replicates shown as points. P-values are from two-sided T-tests. c) HCT116 DNMT3B targets are marked by H3K36me3 in RKO cells. qPCR analysis of H3K36me3 ChIP from RKO cells. The bars indicate the mean of 2 biological replicates with the individual replicates shown as points. d) DNMT3B localises to H3K36me3 marked CGIs in RKO cells. qPCR analysis of T7-tagged DNMT3B from ectopically expressing RKO cells and non-expressing cells as a negative control. The bars indicate the mean of 2 biological replicates with the individual replicates shown as points. Source data are provided as a Source Data file.

Supplementary Figure 4

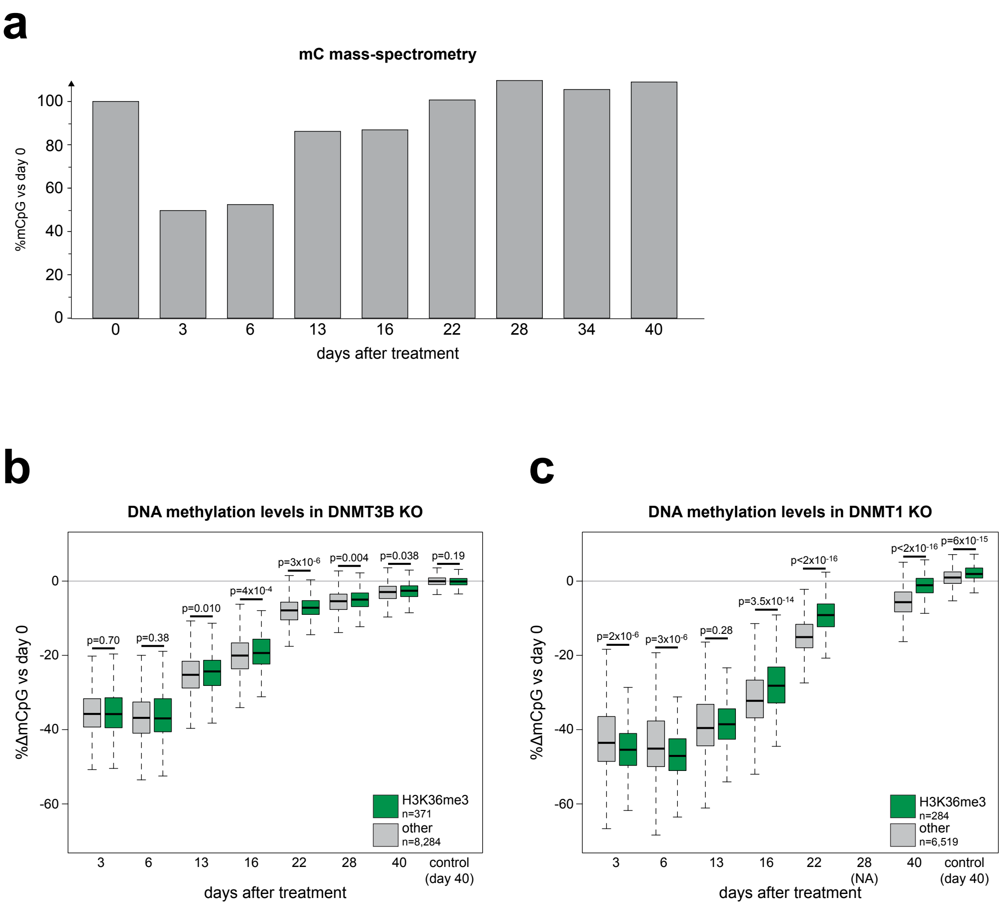

Supplementary Figure 4

H3K36me3 marked CGIs preferentially recover methylation following pharmacological hypomethylation

a) HCT116 DNA methylation levels recover 22 days after 5-aza-dC treatment. Barplot of total methylated cytosine levels estimated by mass-spectrometry. b) Recovery of DNA methylation in DNMT3B KO cells. Boxplots of relative methylation at H3K36me3 marked CGIs and all other CGIs methylated in HCT116 cells. P-values are from two-sided Wilcoxon rank sum tests. Lines=median; box=25th–75th percentile; whiskers=1.5× interquartile range from box. c) Recovery of DNA methylation in DNMT1 KO cells. Boxplots of relative methylation at H3K36me3 marked CGIs and all other CGIs methylated in HCT116 cells. P-values are from two-sided Wilcoxon rank sum tests. Lines=median; box=25th–75th percentile; whiskers=1.5× interquartile range from box. The sample at day 28 failed quality control during processing. Source data are provided as a Source Data file.

Supplementary Figure 5

a

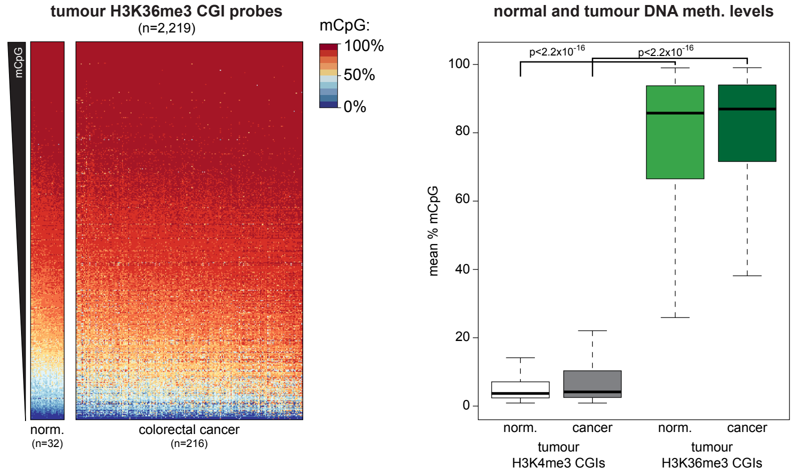

b

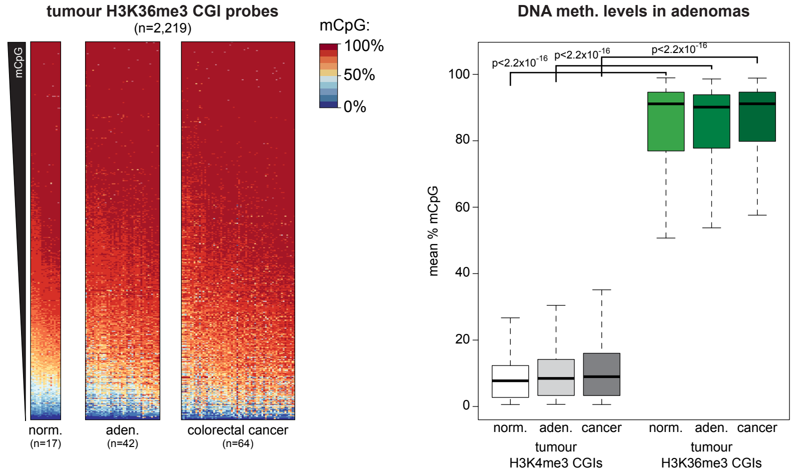

Supplementary Figure 5
H3K36me3 marked CGIs are methylated in colorectal tumours and the normal colon

a) H3K36me3 marked CGIs are methylated in colorectal tumours. Left, Heatmaps of methylation levels at probes located within H3K36me3 peaks and CGIs in a second dataset of colorectal tumours and adjacent normal colon samples. Both CpGs and samples are ordered by mean DNA methylation levels in colorectal tumours. Right, boxplot of the mean CpG methylation for probes located within CGIs and H3K36me3 or H3K4me3 peaks for the same set of samples. P-values are from two-sided Wilcoxon rank sum tests. Lines=median; box=25th–75th percentile; whiskers=1.5× interquartile range from box. norm. = normal colon. b) H3K36me3 marked CGIs are methylated in adenomas. Left, Heatmaps of methylation levels at probes located within H3K36me3 peaks and CGIs in a second dataset of normal colon, adenomas and colorectal tumours. Both CpGs and samples are ordered by mean DNA methylation levels in colorectal tumours. Right, boxplot of the mean CpG methylation for probes located within CGIs and H3K36me3 or H3K4me3 peaks for the same set of samples. P-values are from two-sided Wilcoxon rank sum tests. Lines=median; box=25th–75th percentile; whiskers=1.5× interquartile range from box. norm. = normal colon, aden. = adenoma.

Supplementary Figure 6

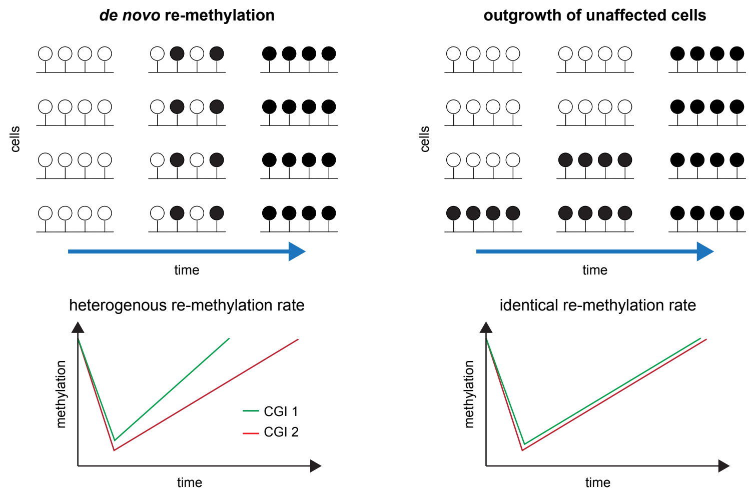

Supplementary Figure 6

Testing whether re-methylation following 5-aza-dC treatment can be explained by the outgrowth of cells escaping hypomethylation. Schematic of the expected dynamics of re-methylation under two different scenarios, de novo methylation or the outgrowth of cells escaping hypomethylation due to a fitness advantage. If re-methylation was entirely explained by outgrowing cells, then the rate of re-methylation relative to the initial methylation level is expected to be identical for each CGI. The upper panel depicts a schematic of methylation levels at a single CGI in a population of cells over time under both models. The lower panel depicts the expected dynamics of re-methylation at different CGIs over time.

Supplementary Figure 7

a supplementary figure 2e top panel
experiment 1 (EF1α)

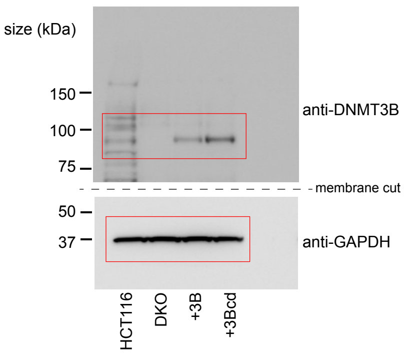

b supplementary figure 2e bottom panel
experiment 2 (CAG, high expr.)

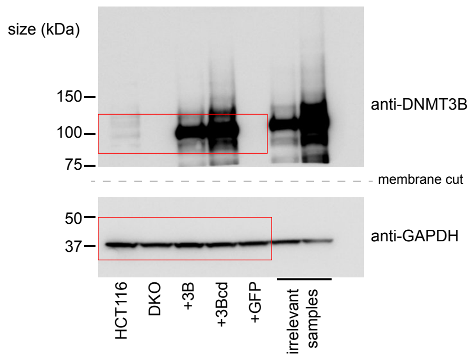

c supplementary figure 2h

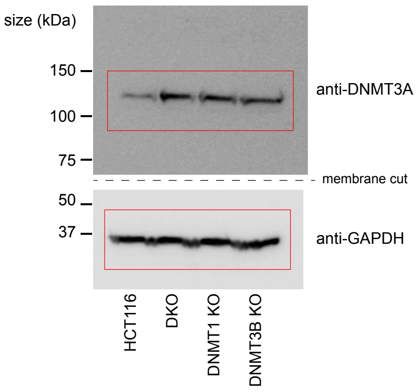

d supplementary figure 2j

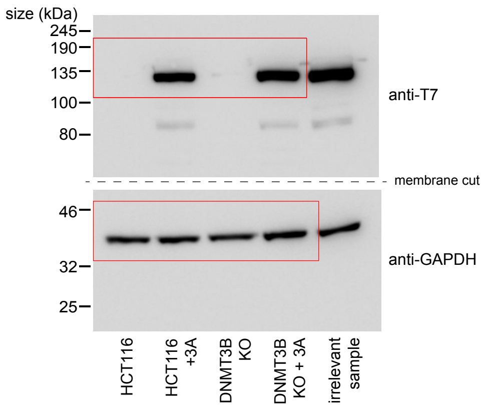

Supplementary Figure 7
Uncropped Western blot scans
Uncropped scans of the Western blots associated with this study. The corresponding figure number is indicated for each blot. Membranes were cut to probe GAPDH loading controls on the same blot as experimental antibodies. Different exposure times were used for the loading control and experimental antibody. Red boxes indicate the part of the membrane shown in the figure. Irrelevant samples were loaded on some gels and not shown in the main figures.
