# Supplementary material for: De novo DNA methyltransferase activity in colorectal cancer is directed towards H3K36me3 marked CpG islands
Source: Nat Commun. 2021 Jan 29;12:694. doi: 10.1038/s41467-020-20716-w (PMC7846778; doi:10.1038/s41467-020-20716-w)
Supplement: Supplementary file 3 — Reporting Summary [file 41467_2020_20716_MOESM3_ESM.pdf]

nature research

Corresponding author(s): Duncan Sproul
Last updated by author(s): Nov 24, 2020

Reporting Summary

Nature Research wishes to improve the reproducibility of the work that we publish. This form provides structure for consistency and transparency in reporting. For further information on Nature Research policies, see our Editorial Policies and the Editorial Policy Checklist.

Statistics

For all statistical analyses, confirm that the following items are present in the figure legend, table legend, main text, or Methods section.

n/a	Confirmed	
☐	☒	The exact sample size (n) for each experimental group/condition, given as a discrete number and unit of measurement
☐	☒	A statement on whether measurements were taken from distinct samples or whether the same sample was measured repeatedly
☐	☒	The statistical test(s) used AND whether they are one- or two-sided<br>*Only common tests should be described solely by name; describe more complex techniques in the Methods section.*
☒	☐	A description of all covariates tested
☐	☒	A description of any assumptions or corrections, such as tests of normality and adjustment for multiple comparisons
☐	☒	A full description of the statistical parameters including central tendency (e.g. means) or other basic estimates (e.g. regression coefficient) AND variation (e.g. standard deviation) or associated estimates of uncertainty (e.g. confidence intervals)
☐	☒	For null hypothesis testing, the test statistic (e.g. F, t, r) with confidence intervals, effect sizes, degrees of freedom and P value noted<br>*Give P values as exact values whenever suitable.*
☒	☐	For Bayesian analysis, information on the choice of priors and Markov chain Monte Carlo settings
☒	☐	For hierarchical and complex designs, identification of the appropriate level for tests and full reporting of outcomes
☐	☒	Estimates of effect sizes (e.g. Cohen's d, Pearson's r), indicating how they were calculated

Our web collection on statistics for biologists contains articles on many of the points above.

Software and code

Policy information about availability of computer code

Data collection: No software was used to collect the data.

Data analysis:
Sequencing data analysis:
BEDtools v2.27.1
FASTQC v0.11.4
Trim Galore v0.4.1
Bowtie2 v2.3.1 and 2.2.6
SAMBAMBA v0.5.9
SAMtools v1.6
Deeptools v3.2.0
Homer v4.8
R v3.4.2 (packages: GO.db v3.4.1, GOSemSim v2.14.2, org.Hs.eg.db 3.11.4, clusterProfiler v3.16.1, lme4 v1.1, minfi v1.22.1)
bcl2fastq v2.17.1.14
NuGen commericial scripts: trimRRBSdiversityAdaptCustomers.py v1.11; nudup.py v2.3

Locus specific bisulfite sequencing: BISMA; BiQ (only one version of each available)
Nucleotide mass-spectrometry: Xcalibur v2.5-204201/2.5.0.2042

For manuscripts utilizing custom algorithms or software that are central to the research but not yet described in published literature, software must be made available to editors and reviewers. We strongly encourage code deposition in a community repository (e.g. GitHub). See the Nature Research guidelines for submitting code & software for further information.

Data

Policy information about availability of data

All manuscripts must include a data availability statement. This statement should provide the following information, where applicable:

- Accession codes, unique identifiers, or web links for publicly available datasets
- A list of figures that have associated raw data
- A description of any restrictions on data availability

All sequencing data generated as part of this study are deposited in NCBI GEO accession GSE158406.

The following previously published datasets were also used:
NCBI's GEO accession: GSE48684
Array Express: E-MTAB-7036

Field-specific reporting

Please select the one below that is the best fit for your research. If you are not sure, read the appropriate sections before making your selection.

☒ Life sciences ☐ Behavioural & social sciences ☐ Ecological, evolutionary & environmental sciences

For a reference copy of the document with all sections, see nature.com/documents/nr-reporting-summary-flat.pdf

Life sciences study design

All studies must disclose on these points even when the disclosure is negative.

Sample size	For experiments, no statistical method was used to pre-determine sample size. Most experiments were based on molecular biology approaches which produce large effect sizes so the sample size was generally 1. We however, made sure to repeat experiments as defined below to enable experimental and biological variability to be ascertained. For the analyses of tissue samples we used all the available data (TCGA= 42 normal, 342 tumours; Fennell et al= 32 normal, 216 tumours; Luo et al= 17 normal, 42 adenoma, 64 tumous).
Data exclusions	No data was excluded.
Replication	All attempts at replication were successful and detailed included in the manuscript. Briefly, the replicated experiments and analyses are:<br>- measurement of DNA methylation by mass spectrometry in knockout cell lines, 3x technical replicates<br>- DNMT3B rescue in DKO cells, replicated twice with different promoters<br>- H3K36me3 ChIP-Rx-seq, 2 biological replicates<br>- DNMT3A ChIP, 3 biological replicates<br>- DNMT3B ChIP-PCR in T7-DNMT3B cells, 3 biological replicates<br>- DNMT3B ChIP-Rx-seq in T7-DNMT3B cells, 2 biological replicates<br>- H3K36me3 ChIP-PCR in RKO cells, 2 biological replicates<br>- DNMT3B ChIP-PCR in RKO cells, 2 biological replicates<br>- piggyBAC experiment and DNMT3B ChIP were also independently replicated in HCT116 and RKO cells<br>- we verified our findings in colorectal tumours in 3 independent datasets.
Randomization	No methods of randomization were used as we used internal controls in experiments to ensure bias was not a significant risk of the experimental design.
Blinding	The investigators were not blinded for collection of experimental data. All data were collected by automated methods with internal controls so investigator judgment could not influence the results.

Reporting for specific materials, systems and methods

We require information from authors about some types of materials, experimental systems and methods used in many studies. Here, indicate whether each material, system or method listed is relevant to your study. If you are not sure if a list item applies to your research, read the appropriate section before selecting a response.

Materials & experimental systems

n/a	Involved in the study
[ ]	[x] Antibodies
[ ]	[x] Eukaryotic cell lines
[x]	[ ] Palaeontology and archaeology
[x]	[ ] Animals and other organisms
[x]	[ ] Human research participants
[x]	[ ] Clinical data
[x]	[ ] Dual use research of concern

Methods

n/a	Involved in the study
[ ]	[x] ChIP-seq
[x]	[ ] Flow cytometry
[x]	[ ] MRI-based neuroimaging

Antibodies

Antibodies used

rabbit anti-DNMT3B (Cell Signalling D7070 lot 1. Cat No: 67259), rabbit anti-DNMT3A (Cell Signalling 2160 lot 2 Cat No: 2160), rabbit anti-GAPDH (Cell Signalling 14C10 lot 10 Cat No: 2118), rabbit anti-T7 (Cell Signalling D9E1X lot 1 Cat No: 13246), rabbit anti-H3K36me3 antibody (ab9050, Abcam lot GR3257952-1). HRP conjugated goat anti-rabbit IgG (Invitrogen, A16110).

Validation

DNMT3A and DNMT3B antibodies were validated by the analysis of siRNA knockdown and knockout cells human HCT116 cells respectively (DNMT3A data not included in study, DNMT3B demonstrated in Supplementary Figure 2e). T7 antibody was validated by the absence of bands in a cell line lacking the tag (demonstrated in Supplementary Figure 2j). GAPDH antibody was not formally validated in this study. H3K36me3 antibody was validated by Western blot in a knockout mouse embryonic stem cell line lacking the enzyme responsible for its deposition, Setd2 (data not included in study). The H3K36me3 mark and its surrounding residues are conserved between mouse and human.

Eukaryotic cell lines

Policy information about cell lines

Cell line source(s)

HCT116 cells and DNMT knockout derivatives were gifts from B. Vogelstein who was the original creator of these cell lines. HCT116 cells carrying T7-tagged DNMT3B were generated in our laboratory during the course of this study. RKO cells were a gift from Malcolm Dunlop.

Authentication

DNMT knockouts were confirmed by western blot and qRT PCR. T7-tagging of DNMT3B was confirmed by Sanger sequencing and Western blot. HCT116 and RKO cells were not formally validated for this study.

Mycoplasma contamination

All cell lines tested negative for mycoplasma.

Commonly misidentified lines
(See ICLAC register)

No commonly misidentified cell lines were used in this study.

ChIP-seq

Data deposition

[x] Confirm that both raw and final processed data have been deposited in a public database such as GEO.

[x] Confirm that you have deposited or provided access to graph files (e.g. BED files) for the called peaks.

Data access links
May remain private before publication.

NCBI GEO: GSE158406

Files in database submission

Files submitted to public repositories are as follows, for 16 samples:
raw FASTQ files
bigWig files

Genome browser session
(e.g. UCSC)

Not available

Methodology

Replicates

Two independent biological replicates were conducted for each experiment.
Correlation coefficients for biological replicate datasets at CGIs analysed in this study (Spearman's Rho):
HCT116 H3K36me3: 0.520
DKO H3K36me3: 0.488
HCT116 T7 (-ve control): -0.080
T7-DNMT3B T7: 0.335

Sequencing depth

All sequencing depths given as million reads or fragments

H3K36me3 ChIP-Rx-seq (75bp single end reads):
HCT116 Input rep. 1, total: 70.54, mapped(hg38,dm6): 59.19/0.11
HCT116 H3K36me3 rep. 1, total: 70.07 58.09/0.29
DKO Input rep.1, total: 72.20 60.76/0.12
DKO H3K36me3 rep. 1, total: 62.24 55.08/0.22
HCT116 Input rep. 2, total: 72.15 60.51/0.13
HCT116 H3K36me3 rep. 2, total: 70.96 58.78/0.28
DKO Input rep.2, total: 74.59 62.69/0.14
DKO H3K36me3 rep. 2, total: 72.79 60.51/0.24

T7-DNMT3B ChIP-Rx-seq (75bp paired end reads):
HCT116 Input rep. 1, total: 74.80, mapped(hg38,dm6): 63.42/0.005
HCT116 T7-IP rep. 1, total: 52.35, mapped(hg38,dm6): 39.36/1.902
T7-DNMT3B Input rep. 1, total: 78.31, mapped(hg38,dm6): 66.45/0.005
T7-DNMT3B T7-IP rep. 1, total: 73.32, mapped(hg38,dm6): 62.90/0.332
HCT116 Input rep. 2, total: 74.94, mapped(hg38,dm6): 63.58/0.006
HCT116 T7-IP rep. 2, total: 66.10, mapped(hg38,dm6): 56.25/0.085
T7-DNMT3B Input rep. 2, total: 68.89, mapped(hg38,dm6): 57.81/0.006
T7-DNMT3B T7-IP rep. 2, total: 83.90, mapped(hg38,dm6): 70.50/0.179

Antibodies

Rabbit anti-H3K36me3 antibody (ab9050, Abcam lot GR3257952-1)
Rabbit anti-T7 (Cell Signalling D9E1X lot 1)

Peak calling parameters

Peak calling was not used for the ChIP-seq data generated in this study.

Data quality

Both sets of data were validated by qPCR. Visual inspection of HCT116 H3K36me3 data used to confirm high degree of correspondence with ENCODE HCT116 H3K36me3 ChIP-seq.

Software

FASTQC v0.11.4
Trim Galore v0.4.1
Bowtie2 v2.3.1
SAMBAMBA v0.5.9
SAMtools v1.6
Deeptools v3.2.0
Homer v4.8
